# Altered gut mycobiome in patients with end-stage renal disease and its correlations with serum and fecal metabolomes

**DOI:** 10.1186/s12967-024-05004-1

**Published:** 2024-02-25

**Authors:** Yi Ren, Lei Chen, Ruochun Guo, Shiyang Ma, Shenghui Li, Yue Zhang, Hongli Jiang, Haitao Shi, Pan Zhang

**Affiliations:** 1 Department of Critical Care Nephrology and Blood Purification, the First Affiliated Hospital of Xi’an Jiaotong University, 277 West Yanta Road, Xi’an, 710061 Shaanxi People’s Republic of China; 2 Department of Nephrology, People’s Hospital of Longhua, Shenzhen, 518109 People’s Republic of China; 3 Department of Gastroenterology, The Second Affiliated Hospital of Xi’an Jiaotong University, No. 157 Xiwu Road, Xi’an, 710004 Shaanxi People’s Republic of China; 4 Shaanxi Key Laboratory of Gastrointestinal Motility Disorders, Xi’an, 710004 Shaanxi People’s Republic of China; 5 Shaanxi Provincial Clinical Research Center for Gastrointestinal Diseases, Xi’an, 710004 Shaanxi People’s Republic of China; 6 Digestive Disease Quality Control Center of Shaanxi Province, Xi’an, 710004 People’s Republic of China; 7 Puensum Genetech Institute, Wuhan, 430076 People’s Republic of China

**Keywords:** End-stage renal disease, Gut mycobiome, Shotgun metagenome sequencing, Serum metabolome, Fecal metabolome

## Abstract

**Background:**

The relationship between the gut mycobiome and end-stage renal disease (ESRD) remains largely unexplored.

**Methods:**

In this study, we compared the gut fungal populations of 223 ESRD patients and 69 healthy controls (HCs) based on shotgun metagenomic sequencing data, and analyzed their associations with host serum and fecal metabolites.

**Results:**

Our findings revealed that ESRD patients had a higher diversity in the gut mycobiome compared to HCs. Dysbiosis of the gut mycobiome in ESRD patients was characterized by a decrease of *Saccharomyces cerevisiae* and an increase in various opportunistic pathogens, such as *Aspergillus fumigatus*, *Cladophialophora immunda*, *Exophiala spinifera*, *Hortaea werneckii*, *Trichophyton rubrum*, and others. Through multi-omics analysis, we observed a substantial contribution of the gut mycobiome to host serum and fecal metabolomes. The opportunistic pathogens enriched in ESRD patients were frequently and positively correlated with the levels of creatinine, homocysteine, and phenylacetylglycine in the serum. The populations of *Saccharomyces*, including the HC-enriched *Saccharomyces cerevisiae*, were frequently and negatively correlated with the levels of various toxic metabolites in the feces.

**Conclusions:**

Our results provided a comprehensive understanding of the associations between the gut mycobiome and the development of ESRD, which had important implications for guiding future therapeutic studies in this field.

**Supplementary Information:**

The online version contains supplementary material available at 10.1186/s12967-024-05004-1.

## Introduction

Chronic kidney disease (CKD) is a long-term progressive renal injury that impacts more than 800 million individuals worldwide [1]. At the end stage of CKD, known as ESRD, patients often require renal replacement therapy, such as dialysis or transplantation, which not only imposes a significant economic burden but also leads to a dramatic fall in their quality of life [2]. This highlights the importance of research into the pathogenesis and treatment of CKD. CKD can have various etiologies, including diabetes, hypertension, smoking, and other underlying conditions [3]. In recent years, the role of gut bacteria in the development of CKD has received considerable attention [4, 5]. A multi-omics study reported that toxins produced by bacteria accumulate in the blood of CKD patients, exacerbating the progression of the disease [5]. Specifically, *Eggerthella lenta* and *Fusobacterium nucleatum* can accelerate the accumulation of phenylacetylglycine, phenyl sulphate, and indoxyl sulphate in the blood of CKD mouse models, leading to an increase in the severity of glomerulosclerosis and renal fibrosis [5]. Several studies have also established a correlation between gut bacteria and CKD clinical characteristics such as diabetes, proteinuria, elevated levels of inflammatory cytokines, and increasing galactose-deficient IgA1 [6–8].

The gut is also a major reservoir of fungi in the human body. Recent studies have highlighted the association between alterations in the gut fungal community and immune-related disorders such as rheumatoid arthritis [9], multiple sclerosis [10], and inflammatory bowel disease [11]. In the context of CKD, patients with impaired immune function and prolonged use of immunosuppressive drugs are particularly susceptible to fungal infections [12]. Importantly, the gut fungal community in CKD patients has been linked to their immunological profiles [13], suggesting a possible influence of gut fungi on immune dysfunction in CKD patients. On the other hand, the impaired intestinal barrier in CKD patients contributed to the translocation of gut microbes or their toxins into the bloodstream [14]. A study with uremic mice indicated that intestinal mucosal injury may exacerbate the translocation of *Candida albicans* and result in systemic infection, although such cases have not been reported in humans [15]. Overall, accumulating evidences hint that the gut mycobiome may play a role in the health of CKD patients.

Currently, the relationship between the gut mycobiome and chronic kidney disease especially ESRD, remains largely unexplored. Given that ESRD patients have a higher risk of fungal infections, we performed a multi-omics analysis based on the gut mycobiome, fecal metabolome, and serum metabolome datasets from 69 healthy controls and 223 ESRD patients. The analysis aimed to identify the gut fungal markers associated with ESRD, and investigate their interactions with host metabolism.

## Methods

### Data sources

All fecal metagenomic sequencing samples from 223 ESRD patients and 69 healthy individuals used in this study are available at the NCBI Sequence Read Archive under the accession ID PRJNA449784 [5]. The serum and fecal metabolomic profiles of all subjects were deposited in the MetaboLights database under the accession ID MTBLS700. The demographic data (e.g., gender, age, and body mass index [BMI]) of subjects were obtained at the following site: https://www.ebi.ac.uk/metabolights/editor/MTBLS700/samples.

### Construction of gut fungi genome catalog

In order to construct a reliable and high-quality catalog of fungal genomes associated with various human body sites, we conducted a comprehensive search in the National Center of Biotechnology Information (NCBI) RefSeq genome database that included approximately 6000 fungal genomes available until April 2020. Subsequently, we manually extracted candidate genomes based on their metadata records in the BioSample or original studies. The extracted genomes had to meet the following criteria: (1) the genome size < 100 Mb and N50 length > 20 kb, (2) documentation of the relevant species colonizing or infecting a specific human body site, and (3) exclusion of non-diet derived fungi such as *Agaricus bisporus*, *Auricularia auricula-judae*, *Ganoderma lucidum*, and so on. A total of 1503 human-associated genomes were retained and clustered into 106 nonredundant genome species-level clusters (hereinafter referred to as “species”) using dRep v3.4.0 with the parameters 'dereplicate -pa 0.9 -sa 0.96 -nc 0.3 -S_algorithm fastANI' [16]. For each fungal species, the genome with the longest N50 length was designated as the reference genome. Finally, the genomes of 106 species were employed to construct our catalog of gut fungal references.

### Processing of metagenomic sequencing data

To ensure data quality, we employed fastp v0.20.164 to process each metagenomic sample [17]. The raw reads suffered from several filtering steps, including trimming of polyG tails and removal of low-quality reads as follows: (1) reads shorter than 90bp; (2) reads with a mean Phred quality score lower than 20; (3) reads with over 30% of their bases having a Phred quality score lower than 20; (4) reads with a mean complexity below 30%; and (5) unpaired-end reads. To minimize the impact of non-specific mapping of reads to fungal genomes in subsequent analysis, we mapped the quality-filtered reads against three databases: the GRCh38 genome, the Unified Human Gastrointestinal Genome (UHGG) collection [18], and the SILVA rRNA database [19]. This step allowed us to exclude reads derived from human or prokaryotic sources.

For each sample, the remaining reads were aligned against our customized catalog of gut fungal genomes using bowtie2 [20], and the read counts for each genome were calculated. To generate mycobiome composition profiles, the read count of each genome was first normalized by dividing its genomic size, and the normalized read count was further divided by the sum of all normalized read counts in a sample. This process defined the relative abundance of each population in the sample. For different fungal taxa, the relative abundance of a taxon was calculated as the sum of the relative abundance of all populations assigned into that taxon.

### Statistical analysis and visualization

Statistical analysis and visualization were carried out by the R language (version 4.1.2) [21].

#### Multivariate analyses

A Bray–Curtis distance matrix was generated using the square-root transformed species-level profiles. This was done using the '*vegdist*' function from the *vegan* package [22]. Principal coordinates analysis (PCoA) was then performed on the distance matrix using the '*pcoa*' function in the *ape* package. Permutational multivariate analysis of variance (PERMANOVA) was conducted using the '*adonis*' function in the *vegan* package, based on the distance matrix. In order to avoid the impact of intra-individual variation, the additional PERMANOVA analysis was performed using the '*adonis*' function with the formula 'Matrix ~ gender + age + BMI + ESRD_status'.

#### Alpha diversity

We calculated the number of observed species by counting the species with a relative abundance greater than zero in each sample. Shannon’s index and Simpson’s index were defined using the function ‘*diversity*’ in the *vegan* package.

#### Significance test

The Wilcoxon rank-sum test was implemented using the function ‘*wilcox.test*’. The student's *t*-test was implemented using the function ‘*t.test*’.

#### Linear discriminant analysis effect size (LEfSe) analysis

Based on the taxonomic profiles combining all taxonomic levels, LEfSe analysis was implemented using the LEfSe Conda version 1.1.01 [23].

#### Explanatory power

According to Wang et al.’s study [5], the evaluation of explanatory power between different omics datasets was performed using stepwise PERMANOVA analysis. For example, in the assessment of the explanatory power of the gut mycobiome on the serum metabolome, the following steps were followed: (1) The R-squared value (R^2^) for each fungal species with respect to the serum metabolome was calculated using the '*adonis*' function, and the resulting R^2^ was adjusted using the '*RsquareAdj*' function. (2) The fungal species exhibiting the largest adjusted R^2^ was selected as the first variate. (3) A second PERMANOVA analysis was executed using the first variate and each remaining fungal species as variables. (4) If the largest adjusted R^2^ from the second PERMANOVA analysis is smaller than that from the first PERMANOVA analysis, the latter is considered as the explanatory power of the gut mycobiome on the serum metabolome. (5) If the largest adjusted R^2^ from the second PERMANOVA analysis is greater, the process is repeated for a third PERMANOVA analysis. In this case, the analysis includes another fungal species in addition to the two variates with the largest adjusted R^2^ from the second PERMANOVA analysis. (6) The process continues until the largest adjusted R^2^ from the last PERMANOVA analysis is smaller than that from the previous PERMANOVA analysis. The latter is then considered as the explanatory power of the gut mycobiome on the serum metabolome.

#### Correlation analysis

We performed a correlation analysis between the relative abundance of gut fungal species and the level of host metabolites using the function ‘*cor.test*’ with the option ‘method = spearman’. The resulting p-values were then adjusted using the function ‘*p.adjust*’ with the option ‘method = BH’. A correlation was considered significant if the adjusted p-value was less than 0.05.

#### Visualization

The sunburst diagram of taxonomic hierarchy was generated using the function ‘*plot_ly*’ in the package *plotly*. All other data were visualized using the function ‘*ggplot*’ in the package *ggplot2*.

#### Classification model

The random forest classifier based on the gut mycobiome was built using the ‘*randomForest*’ function followed by 5 times of five-fold cross-validations, and their performances were evaluated based on area under the receiver operator characteristic curve (AUC) that was calculated by the ‘*roc’* function. The importance ordering of markers was obtained via the ‘*importance*’ function.

## Results

### Sample information and fungal database

In our study, we aimed to characterize the gut mycobiome in patients with ESRD. To achieve this, we performed a re-analysis of publicly available deep-sequencing metagenomic samples. The dataset included samples from 223 ESRD patients and 69 healthy controls (Additional file 1: Table S1), with an average high-quality read data of 11.2 ± 1.7 Gb. The demographic data showed a significant difference between healthy controls and ESRD patients in age and gender (Student's *t*-test, p < 0.001) but not in BMI (Student's *t*-test, *p* = 0.967). More subject metadata had been summarized in Wang’s study [5]. In addition, we obtained serum and fecal metabolome profiles for 284 of the same subjects, allowing us to explore the potential association between the gut mycobiome and the development of ESRD.

**Fig. 1:**
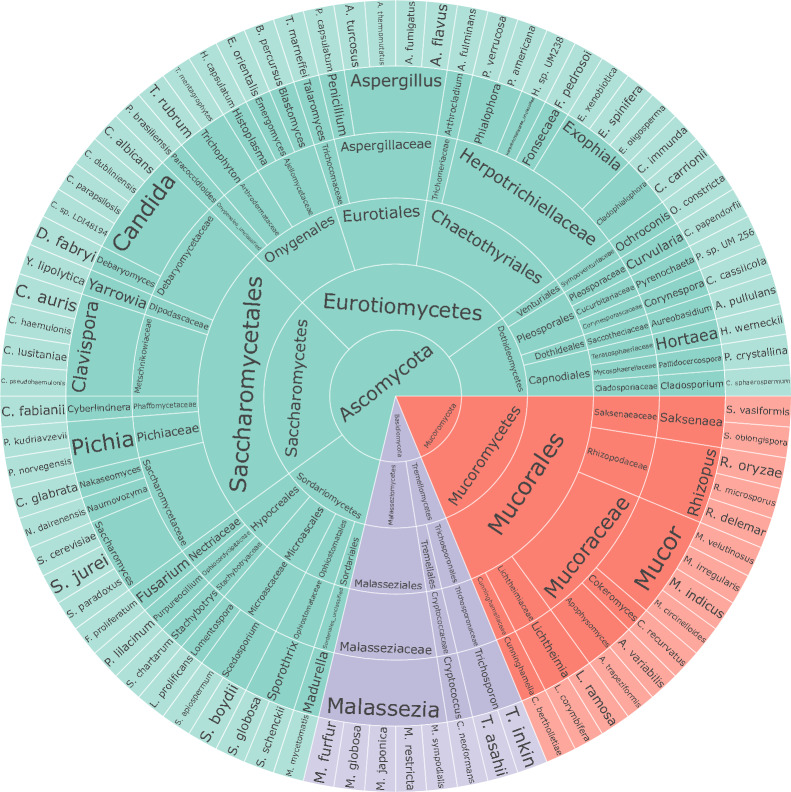
Sunburst diagram of taxonomic hierarchy for 106 gut fungal species and 80 high-level taxa

To accurately determine the composition of the mycobiome, we developed a customized fungi database based on a series of rigorous filter criteria (see details in Methods). This database consisted of 106 nonredundant reference species that were clustered based on a threshold of 96% average nucleotide identity (ANI) from a pool of 1503 human-associated genomes (Additional file 1: Table S2). Subsequently, the high-quality reads from each sample were mapped against the genomes of 106 nonredundant species in the database to generate mycobiome profiles. Besides, 80 high-level taxa were detected in the study samples, representing 48 genera, 35 families, 16 orders, 7 classes, and 3 phyla (Fig. 1).

### Altered gut mycobiome structure in ESRD patients

**Fig. 2:**
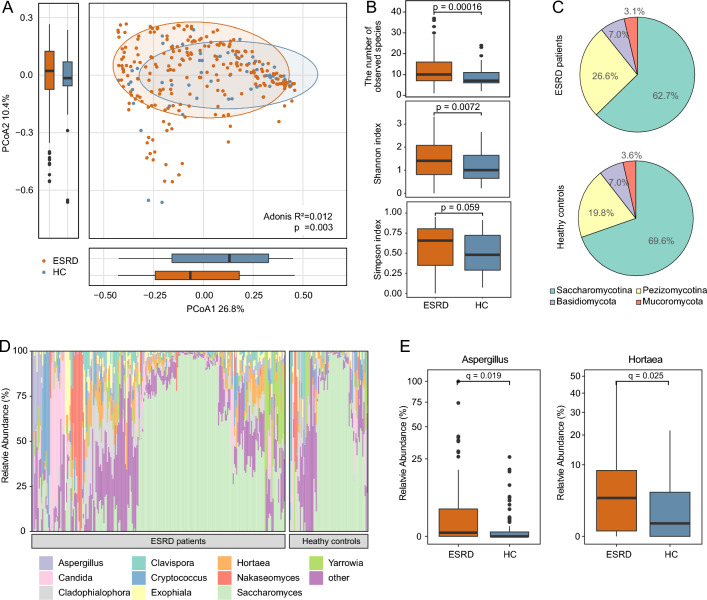
Comparison of gut mycobiome diversity and structure between ESRD patients and healthy controls. **A** PCoA based on Bray–Curtis distance of the fungal profiles at the species level. The plot displayed the distribution of samples along PCoA1 and PCoA2, with ellipsoids indicating the 80% confidence interval for each group. The bottom and left boxplots displayed the sample scores in PCoA1 and PCoA2. **B** Comparison of alpha diversity indexes between ESRD patients and healthy controls. The *p*-value was determined by the Wilcoxon rank-sum test. **C** Pie chart showing the composition of fungal subphyla in each group. The percentages represented the average relative abundance of each subphylum. **D** Distribution of the top 10 abundant genera across all samples. **E** Boxplots showing the relative abundances of the genera Aspergillus and Hortaea in each group. Statistical significance was determined using the Wilcoxon rank-sum test with Benjamini and Hochberg adjustment

We first compared the overall composition structure of the gut mycobiome between healthy controls and ESRD patients using PCoA and PERMANOVA. PCoA based on Bray-cutis distance of species-level composition showed that the top two principal coordinate axes (PCoA1 and PCoA2) accounted for 26.8% and 10.4% of the total variation, respectively (Fig. 2a). Along the PCoA1, ESRD patients showed a mild but statistically significant separation from healthy controls (Wilcoxon rank-sum test, *p* = 0.030). PERMANOVA also showed a significant difference in the gut mycobiome between healthy controls and ESRD patients (*Adonis*, *p* = 0.003). Given the potential confounding effect of individual heterogeneity, we performed additional PERMANOVA analyses by controlling for host variables including gender, age, and BMI. The result showed that ESRD status remained significantly associated with gut mycobiome composition (Adonis, *p* = 0.005), highlighting the robustness of this association.

Alpha diversity was used to estimate the richness and evenness of gut mycobiome in ESRD patients based on three indexes including the number of observed species, Shannon’s index, and Simpson’s index. The ESRD patients showed a higher mycobiome richness and evenness compared to healthy controls, although the significant difference was only observed in Shannon’s index (Wilcoxon rank-sum test, *p* = 0.007; Fig. 2b).

In terms of the fungal taxa, the gut mycobiome of all subjects was usually dominated by Saccharomycotina, followed by Pezizomycotina, Basidiomycota, and Mucoromycota (Fig. 2c). At the genus level, *Saccharomyces* was the first most abundant genus, while other common genera, such as *Aspergillus*, *Candida*, and *Nakaseomyces*, had relatively high abundances in both groups (Fig. 2d). Comparison analysis showed that 2 genera were significantly enriched in ESRD patients (Fig. 2e), including *Aspergillus* (Wilcoxon rank-sum test, adjusted *p* = 0.019) and *Hortaea* (adjusted *p* = 0.025).

### Gut fungal signatures associated with ESRD

**Fig. 3:**
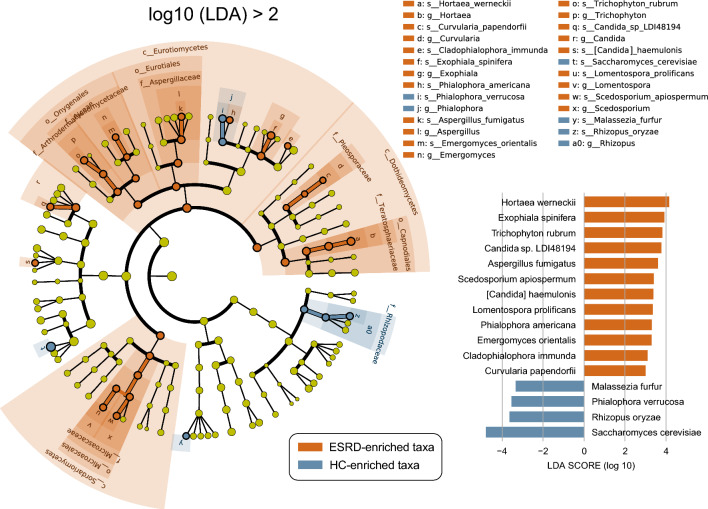
Differential enrichment of gut fungal taxa in ESRD patients and healthy controls. The cladogram visualizes all differentially enriched taxa identified by LEfSe analysis. Each dot corresponds to a fungal taxon, with significant enrichments (*p* < 0.05, LDA > 2.0) labeled in brown for ESRD patients and blue for healthy controls. The bar plot at the bottom right displays the differentially enriched species (*p* < 0.05, LDA > 2.0)

We conducted the LEfSe analysis to identify the fungal taxa that showed statistical differences (p < 0.05, LDA > 2.0) in relative abundance between the ESRD patients and healthy controls. The analysis revealed a total of 41 significantly different fungal taxa, spanning across 3 classes, 4 orders, 7 families, 11 genera, and 16 species (Fig. 3; Additional file 1: Table S3). At the class and order levels, all 7 fungal taxa were significantly enriched in ESRD patients, including the classes Dothideomycetes, Eurotiomycetes, and Sordariomycetes as well as the orders Capnodiales, Eurotiales, Microascales, and Onygenales. At the family level, 6 fungal populations, including Ajellomycetaceae, Arthrodermataceae, Aspergillaceae, Microascaceae, Pleosporaceae, and Teratosphaeriaceae, were significantly enriched in ESRD patients, while only Rhizopodaceae were significantly enriched in healthy controls. At the genus level, 9 fungal populations, including *Aspergillus*, *Candida*, *Curvularia*, *Emergomyces*, *Exophiala*, *Hortaea*, *Lomentospora*, *Scedosporium*, and *Trichophyton*, were significantly enriched in ESRD patients, whereas *Phialophora* and *Rhizopus* were significantly enriched in healthy controls. At the species level, 12 fungal species were enriched in ESRD patients, while 4 species were enriched in healthy controls. Notably, ESRD patients exhibited a significant reduction in the dominant gut fungus *Saccharomyces cerevisiae* compared to healthy controls. Conversely, various opportunistic pathogens were enriched in ESRD patients, including *Aspergillus fumigatus*, *Exophiala spinifera*, *Hortaea werneckii*, *Lomentospora prolificans*, *Trichophyton rubrum*, and so on.

### Correlations between gut mycobiome, serum metabolome, and fecal metabolome

**Fig. 4:**
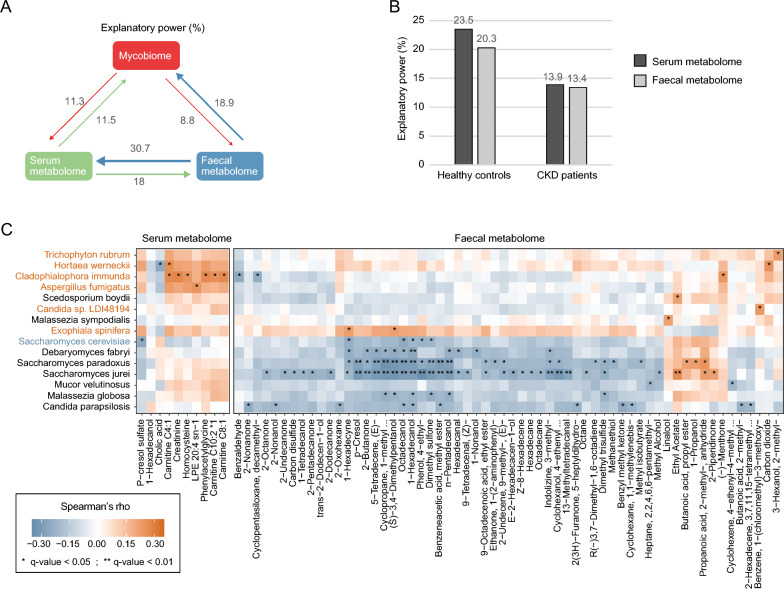
Interaction between the gut mycobiome and host serum and fecal metabolomes. **A** Explanatory power between different omics datasets. The explanatory power was quantified as adjusted R^2^ obtained from stepwise PERMANOVA analysis, as detailed in the Methods. Arrows indicated the direction and magnitude of explanatory power, with numbers indicating the corresponding values. **B** The explanatory power of the gut mycobiome on host serum and fecal metabolomes in ESRD patients and healthy controls. **C** Heatmap displaying the Spearman's rank correlation coefficients between fungal species and host metabolites. Fungal species enriched in ESRD patients were colored in brown, while those enriched in healthy controls were colored in blue. The q-value was obtained using the Benjamini and Hochberg adjustment. Correlations with a q-value < 0.05 were considered statistically significant

In our study, we conducted an integrated analysis of multi-omics datasets, including the gut mycobiome, serum metabolome, and fecal metabolome, to explore the potential contribution of the gut mycobiome to host health. The PERMANOVA analysis indicated that the gut mycobiome explained 11.3% and 8.8% of the variances in the host serum metabolome and fecal metabolome, respectively (Fig. 4a). When differentiating between healthy controls and ESRD patients, both groups show considerable explanatory power of the gut mycobiome for the metabolic profiles. Specifically, the gut mycobiome of ESRD patients accounted for 13.9% and 13.4% of the variance in the serum and fecal metabolome, respectively, while the gut mycobiome of healthy controls accounted for 23.5% and 20.3% of the variance in the serum and fecal metabolome, respectively (Fig. 4b).

To identify the fungal species associated with serum and fecal metabolites, we performed a correlation analysis using Spearman correlation analysis with Benjamini–Hochberg adjustment (q value < 0.05). The results revealed that 15 fungal species were significantly correlated with at least one metabolite, including 6 ESRD-enriched and 1 HC-enriched species (Fig. 4c). Among them, The ESRD-enriched species *Cladophialophora immunda* displayed significant positive correlations with four serum metabolites, namely carnitine, phenylacetylglycine, homocysteine, and creatinine [5]. In the fecal metabolome, *Cladophialophora immunda* showed significant negative correlations with Benzaldehyde and Cyclopentasiloxane. The other 5 ESRD-enriched species, including *Aspergillus fumigatus*, *Exophiala spinifera*, *Hortaea werneckii*, *Trichophyton rubrum*, and *Candida sp. LDI48194*, also exhibited consistent positive associations with the above-mentioned serum metabolites. Conversely, the HC-enriched species *Saccharomyces cerevisiae* was significantly and negatively correlated with three (potential) toxic metabolites, including the serum metabolite p-cresol sulfate, and the fecal metabolites 4-ethylphenol and dimethyl sulfone. Additionally, several fungi that did not show significant differences between healthy controls and CKD patients were frequently associated with fecal metabolites. Particularly, *Saccharomyces paradoxus* and *Saccharomyces jurei* were significantly and negatively correlated with p-cresol, 4-ethylphenol, and dimethyl sulfone in the fecal metabolome.

### Classification of ESRD state based on the gut mycobiome

**Fig. 5:**
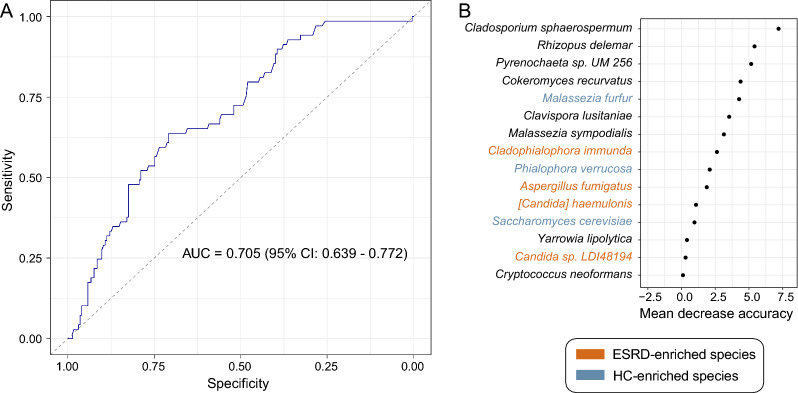
Gut mycobiome-based classification of the ESRD patients and healthy controls. **A** Random forest model for discriminating patients and controls based on gut fungal profiles at the species levels. The area under the receiver-operating characteristic curve (AUC) and 95% confidence interval (CI) are shown. **B** Mean decrease in accuracy of the most 15 important fungal species in the random forest model

Finally, to evaluate the ability of the gut mycobiome to classify ESRD patients and healthy controls, we constructed a random forest model based on the relative abundances of the gut fungal profiles. The model obtained a cross-validation AUC of 0.705 (95% confidence interval [CI] 0.639–0.772; Fig. 5a) in distinguishing patients from controls. Several species, including *Cladosporium sphaerospermum*, *Rhizopus delemar*, control-enriched *Malassezia furfur* and *Phialophora verrucosa*, and ESRD-enriched *Cladophialophora immunda* and *Aspergillus fumigatus* featured the highest discrimination importance in the random forest model (Fig. 5b).

## Discussion

Numerous studies have shed light on the presence of gut dysbiosis in CKD patients, focusing primarily on analyzing the gut bacteriome [5, 24, 25]. However, limited research has been conducted to investigate the relationship between the gut mycobiome and CKD [13]. Here, we constructed a comprehensive gut fungal genome database that closely relates to the human mycobiome. Using this database, we explored the alterations in gut fungal communities in a cohort comprising 69 healthy controls and 223 ESRD patients. Meanwhile, we investigated the relationships between gut fungal species and host fecal and serum metabolites, highlighting the potential impact of gut fungal species on ESRD patients.

We developed a nonredundant gut fungal database comprising 106 species-level genomes. This database exhibited an approximately 25% increase in the number of species compared to the MetaPhlAn 4 database, which included 85 fungal species [26]. Importantly, our database only included fungi that have been reported to colonize or infect various parts of the human body. Fungi derived from dietary sources, such as mushrooms and Ganoderma, were specifically excluded as these fungi are typically transient and lack activity in the gut. Utilizing this database, we observed that *Saccharomyces* was the most prevalent genus in this cohort, which is supported by previous findings [27–29]. Especially, *S. cerevisiae* accounted for 41.5% of the fungal composition.

Microbiome analysis revealed that there were significant differences in fungal community diversity and structure between ESRD patients and healthy controls. We observed a significant increase in alpha diversity indexes among ESRD patients, consistent with a previous study on CKD based on the internal transcribed spacer (ITS) sequencing method [13]. Notably, an increase in gut fungal diversity has been observed in various patient populations with immune system disorders, such as IBD, multiple sclerosis, and acquired immune deficiency syndrome [10, 30, 31]. ESRD patients are characterized by immune dysregulation [12], and their gut fungi have been associated with levels of host C-reactive protein and serum κ/γ light chains [13]. These findings suggest a relationship between the gut mycobiome and the immune system in ESRD patients.

Comparison analysis of fungal community composition revealed 16 species with a significant difference between ESRD patients and healthy controls. Among them, 12 species were enriched in ESRD patients, including *Aspergillus fumigatus*, *Exophiala spinifera*, *Hortaea werneckii*, *Lomentospora prolificans*, and *Trichophyton rubrum*. Fungal infections caused by these pathogens have been previously reported in individuals with kidney disease [32–37]. It is noteworthy that gut *Candida*, particularly *C. albicans*, is enriched in various patient populations with immune, digestive, cancer diseases, and so on [9, 30, 38, 39]. In this study, we also observed a significant increase in *Candida* in ESRD patients. However, at the species level, only *Candida sp. LDI48194* was enriched in ESRD patients, and no significant difference was observed in *C. albicans* between the two groups. This highlights the unique gut fungal dysbiosis in the ESRD population. In contrast, only four species were enriched in the healthy controls, including *Saccharomyces cerevisiae* which was the most abundant fungus in the gut. *Saccharomyces cerevisiae*, a component of the healthy mycobiome, has been reported to possess probiotic properties [30, 40]. Patients with kidney disease often experience some gastrointestinal issues. A previous study has indicated a link between inflammatory bowel disease and an elevated risk of chronic kidney disease [41]. A study in mice has shown that *Saccharomyces cerevisiae* can inhibit and reduce colonic inflammation induced by chemicals [42]. Furthermore, some researchers have developed specific probiotics derived from *Saccharomyces cerevisiae* [43]. These findings hinted the possible potential of S. cerevisiae for improving inflammatory issues in the intestines of kidney disease patients. On the other hand, many articles mention that patients with kidney disease often experience gastrointestinal motility problems, making them prone to constipation [44, 45]. In summary, future intervention studies on Saccharomyces cerevisiae in populations with kidney disease, aiming at improving both intestinal inflammation and constipation, are worth considering.

Besides, the multi-omics analysis revealed a close connection between the gut mycobiome and host metabolome. The previous study has demonstrated that gut bacteria contribute to the accumulation of uremic toxins in the bloodstream of CKD patients [5]. It is worth noting that the explanatory powers of the gut mycobiome on the metabolic profiles were approximately 10% to 20% lower compared to the contribution of the gut bacteriome reported by the previous study [5]. This reduction can be explained by the fact that the gut microbiome is primarily dominated by bacteria, which account for over 80% of the microbial sequences [18]. On the other hand, correlation analysis showed several ESRD-enriched fungi, including *Cladophialophora immunda*, *Aspergillus fumigatus*, and *Hortaea werneckii*, showed a positive correlation with the levels of three uremic toxins (i.e., creatinine, homocysteine, and phenylacetylglycine) in the serum. Conversely, the populations of *Saccharomyces*, including *Saccharomyces cerevisiae*, were found to be significantly and negatively correlated with various (potential) toxic metabolites that were reported to be present in the bloodstream or feces [46–48]. These findings suggest that gut fungi may also play a role in the accumulation of microbiota-driven toxins in the human body.

Our study has some limitations, and it is essential to consider future work to address them. For instance, the absence of longitudinal data in the study hinders our ability to monitor and analyze changes in the gut mycobiome over time in patients with kidney disease. Conducting long-term follow-ups and collecting samples from patients with chronic kidney disease from stages 1 to 5 would enable a systematic exploration of the microbial communities. This is crucial for capturing the dynamic characteristics of the fungal community in the gut and understanding how it responds to various factors or treatments. Moreover, some studies have demonstrated associations between fungal communities and host immune and inflammatory factors. The research by Hu et al. emphasized that the level of serum free light chain lambda in CKD patients was positively correlated with Saccharomyces [13], while Qiu et al. showed a positive association between Saccharomyces and various serum cytokines [49]. Unfortunately, the data we utilized does not include publicly available results of immune globulins or related blood test outcomes [5]. More data is needed to validate the interaction between the immune system and gut fungi in populations with kidney disease.

## Conclusion

In conclusion, our study demonstrates significant changes in the gut mycobiome of ESRD patients which are associated with host immune, inflammation, and toxin levels, ultimately contributing to patient health. However, our study is limited to correlational analyses and does not establish definitive causal relationships. Additionally, due to the lack of detailed clinical information available for the samples, we did not consider other risk factors such as diabetes and hypertension that may influence gut mycobiome. Additional datasets are required to validate the impact of these factors.

### Supplementary Information

**Additional file 1.** **Supplementary Table 1** Sample information from 223 ESRD patients and 69 healthy controls. **Supplementary Table 2** Detailed information of 106 nonredundant genomes. **Supplementary Table 3** Detailed information of the 41 differential taxa identified by LEfSe analysis.

## Data Availability

The datasets used and/or analysed during the current study are available from the corresponding author on reasonable request. All data generated or analysed during this study are included in this published article (and its Additional file 1).
